# Myostatin Knockout Limits Exercise-Induced Reduction in Bovine Erythrocyte Oxidative Stress by Enhancing the Efficiency of the Pentose Phosphate Pathway

**DOI:** 10.3390/ani12070927

**Published:** 2022-04-04

**Authors:** Lin Zhu, Chunling Bai, Xueqiao Wang, Zhuying Wei, Mingjuan Gu, Xinyu Zhou, Guanghua Su, Xuefei Liu, Lei Yang, Guangpeng Li

**Affiliations:** State Key Laboratory of Reproductive Regulation and Breeding of Grassland Livestock, College of Life Science, Inner Mongolia University, Hohhot 010021, China; zhulinynacxhs@163.com (L.Z.); chunling1980_0@163.com (C.B.); wangxueqiao05@126.com (X.W.); weizhuying2008@126.com (Z.W.); gmj0119@yeah.net (M.G.); zhouxinyufjb@163.com (X.Z.); suguanghua0707@163.com (G.S.); liuxuefei1006@126.com (X.L.)

**Keywords:** erythrocytes, myostatin, antioxidants, pentose phosphate pathway

## Abstract

**Simple Summary:**

Myostatin (MSTN) is mainly expressed in skeletal muscle and is involved in the regulation of skeletal muscle growth and development. Loss of MSTN results in muscle hypertrophy. In this study; red blood cells were used as materials to study the effect of MSTN on the antioxidant capacity of bovine erythrocyte after exhaustive exercise. The findings suggest that knockdown of MSTN accelerates the pentose phosphate pathway; thereby enhancing the antioxidant capacity of erythrocytes.

**Abstract:**

Moderate exercise can strengthen the body, however, exhaustive exercise generates large amounts of reactive oxygen species (ROS). Although erythrocytes have antioxidant systems that quickly eliminate ROS, erythrocytes become overwhelmed by ROS when the body is under oxidative stress, such as during exhaustive exercise. Myostatin (MSTN) has important effects on muscle hair development. Individuals lacking myostatin (MSTN) exhibit increased muscle mass. The purpose of this study was to investigate the mechanism by which MSTN affects erythrocyte antioxidant changes after exhaustive exercise in cattle. Antioxidant and metabolite detection analysis, western blotting, immunofluorescence, and fatty acid methyl ester analysis were used to assess exercise-associated antioxidant changes in erythrocytes with or without MSTN. Knockdown of MSTN enhances Glucose-6-phosphate dehydrogenase (G6PD) activity after exhaustive exercise. MSTN and its receptors were present on the erythrocyte membrane, but their levels, especially that of TGF-β RI, were significantly reduced in the absence of MSTN and following exhaustive exercise. Our results suggest that knockout of MSTN accelerates the pentose phosphate pathway (PPP), thereby enhancing the antioxidant capacity of erythrocytes. These results provide important insights into the role of MSTN in erythrocyte antioxidant regulation after exhaustive exercise.

## 1. Introduction

Erythrocytes (i.e., red blood cells) are the primary transporter of oxygen in the blood in vertebrates [1]. This critical function can be impaired by cellular damage caused by reactive oxygen species (ROS). Major cellular ROS include superoxide anion (O_2_^-^), hydrogen peroxide (H_2_O_2_), and hydroxyl radical (OH). The two primary sources of cellular ROS are the mitochondrial electron transport chain [2], and substrate oxidation by NADPH oxidase (NOX) and other oxidases [3]. Mature mammalian erythrocytes are anucleate (i.e., have no DNA) and lack mitochondria [4], instead producing energy by anerobic glycolysis [5]. Accordingly, the primary endogenous sources of erythrocyte ROS are hemoglobin (HGB) autoxidation [6], which occurs slowly but continually [7].

In addition to endogenous ROS, erythrocytes are exposed to exogenous ROS released by neutrophils, macrophages, and endothelial cells into the plasma [8]. Because exogenous ROS are taken up by erythrocytes [9], they affect erythrocyte redox status [10]. To minimize ROS-induced lipid and protein damage [11] and associated oxidative stress [12], erythrocytes have an extensive antioxidant system involving both enzymatic antioxidants, including superoxide dismutase (SOD), catalase (CAT), and peroxidase (POD) [7], and nonenzymatic antioxidants like reduced glutathione (GSH) [12].

Moderate exercise can enhance the body’s immunity against age-related illnesses. However, extreme exercise, such as exercising to exhaustion or under hypoxic conditions, is associated with increased risk for various cardiovascular diseases, or even sudden death [13,14], this is because exercise can increase plasma ROS levels [15], and exhaustive exercise generates a burst of free radicals that causes serious damage to tissues, sometimes leading to multiple organ failure [16]. Exhaustive exercise-induced ROS has been shown to cause erythrocyte oxidative stress. Exhaustive exercise induces dysfunction of erythrocyte deformability, possibly via alterations in the Band 3 protein (also known as anion exchanger 1, AE1) [17]. Oxidative damage manifests as increased protein carbonylation and lipid peroxidation (i.e., increased malondialdehyde [MDA] levels). In humans, acute physical exercise produces decreased erythrocyte membrane fluidity and increased levels of protein carbonyls (PC), although plasma MDA and 4-HAD concentrations do not change significantly [18]. Another study found that exercise induces decreased erythrocyte membrane fluidity in the polar region, alterations in membrane protein conformations, and increased lipid peroxidation, although no free radicals were detected [19]. Additionally, exhaustive exercise significantly increases the activities of CAT and glutathione reductase (GR) and significantly decreases glutathione peroxidase (GSH-Px) activity [20]. In rats, exercise increases erythrocyte concentrations of thiobarbituric acid-reactive substances and PC and decreases the ratio of GSH to glutathione disulfide (GSSG) [21].

Under the condition that GR needs NADPH to provide reducing power, GSSG is reconverted to GSH to maintain the balance of GSH/GSSG, in which the reducing power provided by NADPH generated by the pentose phosphate pathway (PPP) can make GSSG in the cell in a reduced state [22]. G6PD helps protect against oxidative damage in erythrocytes [23], as evidenced by the significantly higher levels of oxidative damage seen in G6PD-deficient erythrocytes [24].

In mature erythrocytes, the antioxidant capacity of erythrocytes is closely related to glycolysis. Erythrocytes have no mitochondria, the energy depends on glycolysis, which uses glucose as a substrate. Following glucose uptake from the plasma, hexokinase (HK) converts glucose to glucose-6-phosphate (G6P), the small part of which enters the PPP, most are broken down by glycolysis into pyruvic acid and lactic acid, and provide ATP to erythrocytes.

Myostatin (MSTN) is a member of the transforming growth factor β superfamily, mutations in the MSTN gene usually caused the ‘double muscled’ phenotype [25]. MSTN is expressed in skeletal muscle most prominently, and also expressed in other tissues [26,27]. MSTN is an energy regulator that mediate cell glucose metabolism. In C2C12 cells, overexpressed MSTN accompanied by reduced GlUT4 gene expression and glucose uptake [28]. Treatment old mice with an anti-myostatin antibody increased whole body glucose metabolism [29]. MSTN inactivation represses glycolysis and glycogen accumulation [30].

Erythrocytes are important oxygen carriers in the blood circulation, providing oxygen for all tissue cells to ensure the efficiency of aerobic exercise. The oxygen carrying capacity of erythrocytes is affected by oxidative damage. However, it remains to be determined whether MSTN knockout affects the antioxidant capacity of erythrocytes.

In this study, we investigated how MSTN knockout affects erythrocyte oxidative damage and antioxidant capacity. Specifically, using an exhaustive exercise model in cattle, we assessed the effect of MSTN knockout on exercise-induced oxidative damage in erythrocytes. We found that exhaustive exercise increased oxidative damage in bovine erythrocytes, and MSTN knockout enhanced the erythrocyte antioxidant capacity by enhancing metabolism through the PPP. These results provide insight into the antioxidant capacity of erythrocytes of MSTN after exhaustive exercise.

## 2. Materials and Methods

### 2.1. Ethics Statement

All experimental procedures used in this research were in accordance with the the Regulation on the Administration of Laboratory Animals (2017, China State Council). All protocols were approved by the Animal Ethics Committee of Inner Mongolia University. This experiment was carried out with the approval of the ethics committee of experimental animals of Inner Mongolia University (No. IMU-CATTLE-2020-033, 1 April 2020).

### 2.2. Animals

As in our previous report, generation of MSTN-knockout Luxi cattle using CRISPR/Cas9 [31,32]. Wild-type and MSTN knockout cattle (10 animals each), 24 months of age, were used in this study. All cattle were raised in the same field and were fed under the same conditions.

The weight, height and body length of MSTN knockout cattle and wild cattle are shown in the Supplementary Tables (Tables S1–S3). Red blood cells were collected from the neck of cattle, and 5 mL EDTA K_2_ blood vessels were collected. The obtained blood was added into a 50 mL centrifuge tube and PBS (PBS: blood = 9:1) was added. Centrifugation at 4 °C for 30 min could obtain red blood cells.

### 2.3. Experimental Design

**Figure 1:**
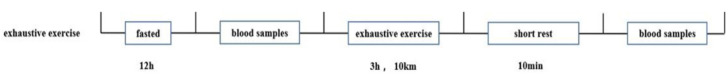
Exhaustive exercise experimental design.

Before exhaustive exercise, cattle were fasted for 12 h, at which point resting-state (RS) blood samples were collected from the jugular vein. For exercise to failure, the cow ran on the ground for 3 h at a moderate speed, covering a distance of 10 km. Cattle were considered exhausted when they would no longer run, even when prodded. After a short rest (10 min), an exhaustive-exercise-state (EE) blood sample was collected from the neck vein. Cattle were subjected to exhaustive exercise once a week for 3 weeks (Figure 1) [33,34,35].

### 2.4. Blood Sample Preparation

Blood samples were poured into EDTA-containing tubes and serum-separator tubes immediately following collection. The EDTA-tubes samples were washed three times with PBS by centrifugation (1500 rpm/min, 15 min, 4 °C), after which the plasma and white blood cells were removed, and the packed erythrocytes were collected. The sera from samples in serum-separator tubes were centrifuged (4000× *g*, 5 min, 4 °C) and supernatants were collected for analysis.

### 2.5. Antioxidant and Metabolite Detection Analysis

Erythrocytes were analyzed by commercial kits following the manufacturer’s instructions. Briefly, 5 × 10^5^ cells were lysed in erythrocyte lysis buffer by sonication (30 cycles, 3 s pulses, 20% power, 10 s intervals). The supernatant was added to a 96-well plate containing the reagents, and well absorbances were measured using a spectrophotometer (Thermo, Waltham, MA, USA). Absorbance values were used to calculate the activities of various enzymes, including SOD (SOD-1-Y,COMIN Biotechnology, Suzhou, China), CAT (CAT-1-Y, COMIN Biotechnology, Suzhou China), GR (GR-1-W, COMIN Biotechnology, Suzhou, China), GSH-Px (GPX-1-W, COMIN Biotechnology, Suzhou, China), HK (HK-1-Y, COMIN Biotechnology, Suzhou, China), G6PD (G6PDH-1-Y, COMIN Biotechnology, Suzhou, China), PK (PK-1-Y, COMIN Biotechnology, Suzhou, China), PFK (PK-1-Y, COMIN Biotechnology, Suzhou, China), POD (POD-1-Y, COMIN Biotechnology, Suzhou, China), MHBR (KS16455, KESHUN Biotechnology, Shanghai, China) and the concentrations of metabolites, including MHB (BH-ELISA3340, BUOHUI Biotechnology, Shanghai, China), NADPH (NADP-1-Y, COMIN Biotechnology, Suzhou, China), GSH (GSH-1-W, COMIN Biotechnology, Suzhou, China), GSSG (GSSG-1-W,COMIN Biotechnology, Suzhou, China), PA (PA-1-Y, COMIN Biotechnology, Suzhou, China) and LA (LA-1-G,COMIN Biotechnology, Suzhou, China), 6PGDH (BC2105, Solarbio, Beijing, China), RPI (ab196994, Abcam, Cambridge, MA, USA).

### 2.6. Fatty Acid (FA) Methyl Ester Analysis

Erythrocytes were washed twice with PBS and mixed with 1 mL of 2.5% (*v/v*) H_2_SO_4_ in methanol. Samples were incubated at 80 °C for 90 min. After cooling to room temperature (RT), 1.5 mL of 0.9% NaCl was added. The samples were mixed, vortexed (5 min), and then centrifuged (2000 rpm/min, 5 min) to isolate the FA-containing organic phase. After transferring the supernatants to fresh tubes, 0.4 mL of a saturated KOH solution in methanol was added, and samples were mixed, vortexed (5 min), and centrifuged (2000 rpm/min, 10 min). The supernatant FA were analyzed using gas chromatography-mass spectroscopy (Shimadzu, Kyoto, Japan).

### 2.7. Western Blotting

Erythrocytes were lysed on ice using Mammalian Protein Extraction Reagent (CWBiotech, Beijing, China), and protein concentrations were determined using Varioskan Flash (Thermo, Waltham, MA, USA). Lysate supernatants were boiled for 10 min, separated on an 10% SDS-polyacrylamide gel, and then transferred to nitrocellulose membranes (Bio-sharp, Beijing, China). Membranes were blocked for 1 h in 5% skim milk in Tris-buffered saline with Tween (TBST) and were then incubated with antibodies against Band 3 (1:1000, Abcam, Cambridge, MA, USA), TGF-β RI (1:1000, Abcam, Cambridge, MA, USA), Act RII (1:1000, Abcam, Cambridge, MA, USA) and α-tubulin (1:2000, Abcam, Cambridge, MA, USA) overnight at 4 °C. Membranes were washed three times with TBST. Membranes were then incubated with peroxidase-conjugated immunoglobulin G antibody (1:5000; Jackson Immunoresearch, Lancaster, PA, USA) for 2 h at RT before washing with TBST. After adding developing liquid, chemiluminescent signals were detected using a Tanon-5200 system (YuanPingHao Biotech, Beijing, China).

### 2.8. MSTN Protein Was Detected by ELISA

The isolated erythrocytes were tested according to the kit is a bovine MSTN ELISA Kit (CSB-EL015057BO, CUSABIO, Wuhan, China) instructions. Remove each reagent to room temperature for 30 min; Add 100 μL standard or sample to each well, and incubate at 37 °C for 2 h. 100 μL biotin-labeled antibody working solution, incubated at 37 °C for 1 h; Wash 3 times, soak for 2 min each, 200 μL/ well, dry by swing; Add 100 μL of pepper root peroxidase labeled avidin working solution to each well, and incubate at 37 °C for 1 h. Wash solution 5 times, soak 2 min each, 200 μL/ well, spin-dry; Add 90 μL substrate solution to each well, and shade at 37 °C for 18 min. Add 50 μL stop solution to each well to stop the reaction. Within 5 min after the termination of the reaction, a microplate reader with a wavelength of 450 nm was used. The optical density (OD) of each well was measured, and the MSTN protein content in the sample was calculated according to the standard curve. The kit detects the mature peptide of MSTN.

### 2.9. Immunofluorescence Staining

Erythrocytes were rinsed three times with PBS, fixed with 4% paraformaldehyde 10 min at RT, permeabilized with 0.01% Triton X-100 for 30 min at RT, and then thoroughly washed with 0.3% BSA in PBS. Fixed samples were blocked with 3% BSA, in PBS at 37 °C for 1 h and then incubated with anti-Band 3 antibody overnight at 4 °C. Samples were then washed several times in PBS and incubated for 1 h at 37 °C with CoraLite594—conjugated Goat Anti-Rabbit IgG (H + L) secondary antibodies (Proteintech, Wuhan, China). Stained erythrocytes were imaged on a laser scanning microscope (Nikon, Tokyo, Japan).

### 2.10. Calculations and Statistical Analyses

All data are expressed as the mean ± standard error of the mean of at least three independent experimental replicates. In graphs, bars represent means, and error bars represent one standard error. Statistical significance was evaluated by two-way ANOVA and Welch’s two-tailed *t*-test with Bonferroni correction for post hoc analysis to adjust for multiple comparisons; *p*-values < 0.05 were considered statistically significant (*, *p* < 0.05; **, *p* < 0.01).

## 3. Results

### 3.1. Effect of Exhaustive Exercise on Bovine Hematological Parameters

**Figure 2:**
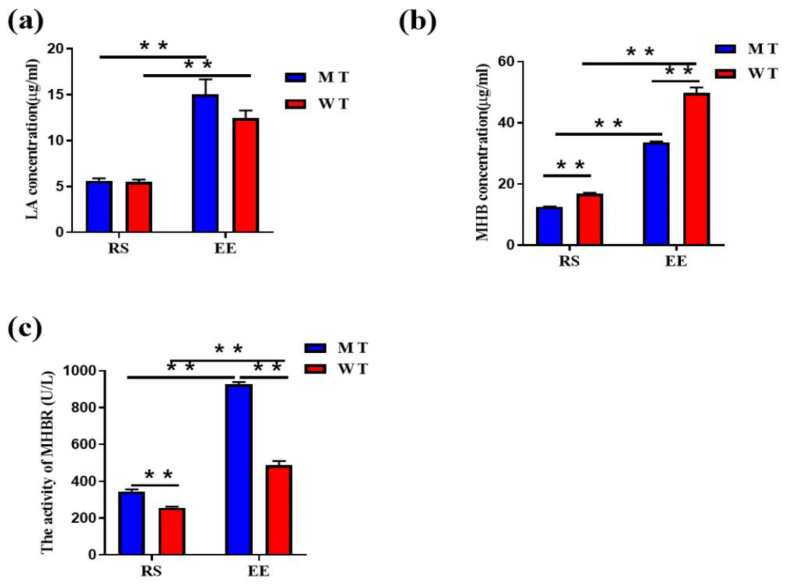
Effect of exhaustive exercise on MSTN knockout cattle. (**a**) Lactic acid concentration in the cattle plasma; (**b**) Levels of MHB; (**c**) The activity of MHBR. MT: MSTN knockout cattle group;WT: Wild type cattle group; RS: Resting state; EE: Exhaustive exercise state; Data presented are means ± SD. One-way ANOVA with post hoc LSD multiple-comparison test, ** *p* < 0.01.

The effects of exhaustive exercise on bovine hematological parameters are shown in Table 1 and Figure 2. There were no significant differences between Wild-type (WT) and MSTN knockou (MT) cattle for any parameter at either resting-state (RS) or Exhaustive exercise (EE). EE caused slight decreases in red cell distribution width, mean corpuscular volume, and haemoglobin (HGB) levels, and a slight increase in hematocrit in both groups (Table 1). Additionally, plasma lactic acid (LA) levels increased dramatically after exhaustive exercise in both MT cattle (RS vs. EE, 5.64 ± 0.24 vs. 15.05 ± 1.62 μg/mL) and WT cattle (RS vs. EE, 5.49 ± 0.24 vs. 12.44 ± 0.84 μg/mL; Figure 2a). The concentration of Methemoglobin (MHB) in MT group was significantly lower than that in WT group at RS (12.54 ± 0.138 vs. 16.73 ± 0.37 μg/mL) and EE (33.62 ± 0.29 vs. 49.83 ± 1.72 μg/mL), and the MHB increased significantly after exhaustive exercise in both MT cattle and WT cattle group (Figure 2b). The Methemoglobin Reductase (MHBR) is closely related to the content of MHB, and was significantly higher in MT group than in WT group both at RS (344.97 ± 11.37 vs. 256.97 ± 5.8 U/L) and EE (927.13 ± 11.522 vs. 488.80 ± 21.65 U/L; Figure 2c). These results indicated that the cattle model of exhaustive exercise was effective, it also indicates that MSTN knockout affects the oxidation of red blood cells.

### 3.2. MT Erythrocytes Exhibit Less Oxidative Damage after Exhaustive Exercise

**Figure 3:**
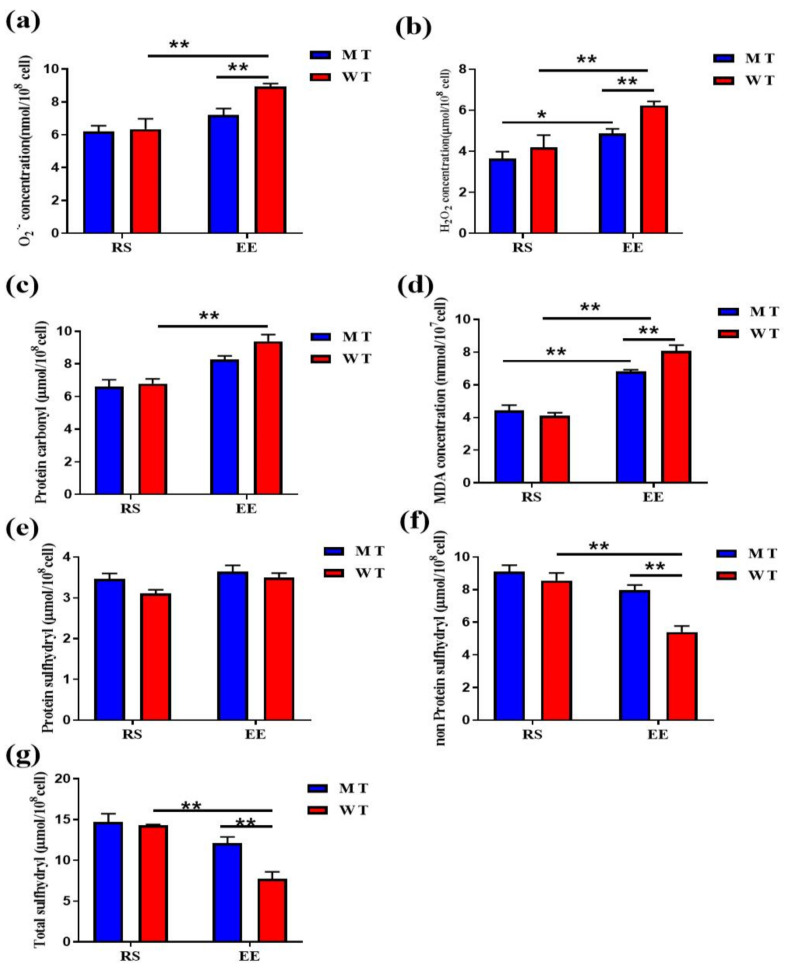
MSTN knockout reduced the oxidative damage of erythrocytes after exhaustive exercise. (**a**) Concentration of O_2_^·^^-^ in the WT and MT cattle erythrocytes; (**b**) Concentration of H_2_O_2_; (**c**) Levels of protein carbonyl; (**d**) MDA concentration; (**e**) Protein sulfhydryl; (**f**) Non-protein sulfhydryl; (**g**) Total sulfhydryl state. MT: MSTN knockout cattle group; WT: Wild type cattle group; RS: Resting state; EE: Exhaustive exercise state. Data presented are means ± SD. One-way ANOVA with post hoc LSD multiple-comparison test. * *p* < 0.05, ** *p* < 0.01.

At the resting state, H_2_O_2_ levels were similar between MT-RS and WT-RS erythrocytes (3.64 ± 0.33 and 4.18 ± 0.66 μmol/10^8^ cells). Exhaustive exercise significantly increased H_2_O_2_ levels in both MT erythrocytes (4.88 ± 0.21 μmol/10^8^ cells) and WT erythrocytes (6.23 ± 0.19 μmol/10^8^ cells), but MT-EE H_2_O_2_ levels remained significantly lower than WT-EE levels (Figure 3a). O_2_^·^^-^ levels were similar between MT-RS and WT-RS erythrocytes (6.19 ± 0.35 and 6.34 ± 0.63 nmol/10^8^ cells, respectively). O_2_^·^^-^ levels were elevated significantly in WT-EE erythrocytes (8.93 ± 0.19 nmol/10^8^ cells) and only slightly in MT-EE erythrocytes (7.20 ± 0.38 nmol/10^8^ cells), with MT-EE O_2_^·^^-^ levels remaining markedly lower than WT-EE levels (Figure 3b). These data show that exhaustive exercise induces elevated ROS levels in both MT-EE and WT-EE erythrocytes.

We next examined if the elevated exercise-induced ROS levels caused erythrocyte protein or lipid damage. Protein carbonyl levels were elevated significantly in WT-EE erythrocytes (RS vs. EE, 6.79 ± 0.29 vs. 9.38 ± 0.41 μmol/10^8^ cells) and increased less markedly in WT-EE erythrocytes (RS vs. EE, 6.59 ± 0.45 vs. 8.27 ± 0.22 μmol/10^8^ cells) (Figure 3c). MDA concentrations were similar in MT-RS erythrocytes (4.44 ± 0.31 μmol/10^7^ cells) and WT-RS erythrocytes (4.11 ± 0.18 μmol/10^7^ cells), and exhaustive exercise increased MDA concentrations in both MT and WT erythrocytes (6.82 ± 0.09 and 8.06 ± 0.36 μmol/10^7^ cells, respectively; RS EE for either type), with concentrations remaining lower in MT-EE erythrocytes than in WT-EE erythrocytes (Figure 3d). Protein sulfhydryl levels were similar in all erythrocytes (MT-RS, 3.46 ± 0.13μmol/10^8^ cells; WT-RS, 3.10 ± 0.08 μmol/10^8^ cells; MT-EE, 3.64 ± 0.14μmol/10^8^ cells; and WT-EE, 3.49 ± 0.10 μmol/10^8^ cells; Figure 3e). Nonprotein sulfhydryl levels decreased significantly in WT erythrocytes after exhaustive exercise (RS vs. EE, 8.55 ± 0.47 vs. 5.40 ± 0.37 μmol/10^8^ cells), whereas they decreased only slightly in MT erythrocytes (RS vs. EE, 9.11 ± 0.39 vs. 7.95 ± 0.32 μmol/10^8^ cells). The exercise-induced variations in total sulfhydryl levels mirrored those of nonprotein sulfhydryls (MT-RS, 14.69 ± 1.01 μmol/10^8^ cells; MT-EE, 12.11 ± 0.75 μmol/10^8^ cells; WT-RS, 14.30 ± 0.11 μmol/10^8^ cells; WT-EE, 7.75 ± 0.83 μmol/10^8^ cells; Figure 3g). These results suggest that knockdown of MSTN partially attenuates exercise-induced erythrocyte production of ROS, PC and lipid peroxides.

FA is an important structural component of erythrocyte membrane and can be destroyed by ROS. After exercise, total FA concentrations in MT and WT erythrocytes decreased, respectively (Figure S1A). MSTN knockdown slightly decreased the SFA content of RS and significantly decreased the SFA content of EE. For UFA, there was no significant difference between MT and WT erythrocytes before and after exercise, and exercise did not affect UFA levels (Figure S1B). However, UFAs include monounsaturated FA (MUFA) and polyunsaturated FA (PUFA), and MUFA concentrations in MT erythrocytes and WT erythrocytes were significantly reduced upon EE. There was no significant difference in PUFA between MT and WT erythrocytes before and after exercise (Figure S1C). C18:3n6 and C20:3n6 concentrations were low in all groups and showed little variation (Figure S1E).

### 3.3. MSTN Knockout Increases the Erythrocyte Antioxidant Capacity

**Figure 4:**
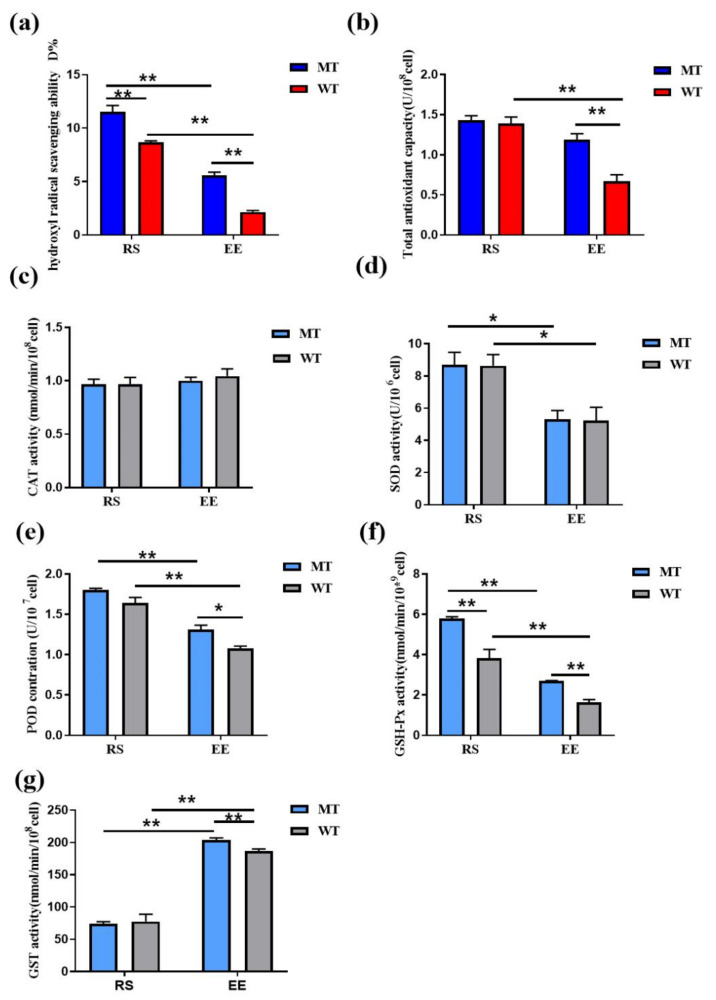
MSTN knockout enhanced the antioxidant capacity of erythrocytes. (**a**) Hydroxyl radical scavenging ability of erythrocytes; (**b**) Total antioxidant capacity of erythrocytes; (**c**) Activity of CAT; (**d**) Activity of SOD; (**e**) Activity of POD; (**f**) Activity of GSH-Px; (**g**) Activity of GST. MT: MSTN knockout cattle group; WT: Wild type cattle group; RS: Resting state; EE: Exhaustive exercise state; Data presented are means ± SD. One-way ANOVA with post hoc LSD multiple-comparison test. * *p* < 0.05, ** *p* < 0.01.

The hydroxyl radical scavenging ability were higher in MT-RS and MT-EE erythrocytes (11.51 ± 0.60% and 5.58 ± 0.29%, respectively) than in WT-RS and WT-EE erythrocytes (8.65 ± 0.15% and 2.6 ± 0.12%, respectively), and exhaustive exercise significantly reduced the activity in both erythrocyte types (Figure 4a). At RS, total antioxidant capacity did not differ between MT and WT erythrocytes (1.43 ± 0.05 and 1.39 ± 0.08 U/10^8^ cells, respectively). Exhaustive exercise significantly reduced total antioxidant capacity in WT erythrocytes (0.67 ± 0.08 U/10^8^ cells; RS vs. EE) but did so only slightly in MT erythrocytes (1.19 ± 0.08 U/10^8^ cells; RS vs. EE), such that MT-EE erythrocytes had higher antioxidant capacity than WT-EE erythrocytes (Figure 4b).

CAT activities were similar in all samples (MT-RS, 0.96 ± 0.04 nmol/min/10^8^ cells; WT-RS, 0.98 ± 0.06 nmol/min/10^8^ cells; MT-EE, 1.00 ± 0.03 nmol/min/10^8^ cells; and WT-EE, 1.04 ± 0.04 nmol/min/10^8^ cells; Figure 4c). Exercise affected SOD activity in both MT erythrocytes (RS vs. EE, 8.68 ± 0.79 vs. 5.30 ± 0.55 U/10^6^ cells) and WT erythrocytes (RS vs. EE, 8.64 ± 0.69 vs. 5.23 ± 0.81 U/10^6^ cells), and MSTN had little effect on SOD activity regardless of exercise (Figure 4d). POD activity mirrored SOD activity, with an exercise-induced reduction in POD activity in both MT erythrocytes (RS vs. EE, 1.80 ± 0.01 vs. 1.31 ± 0.54 U/10^7^ cells) and WT erythrocytes (RS vs. EE, 1.62 ± 0.06 vs. 1.07 ± 0.03 U/10^7^ cells) and partial alleviation of this reduction by MSTN knockout (Figure 4e). MSTN had a more dramatic effect on GSH-Px activity. GSH-Px activity was higher in MT-RS erythrocytes (5.78 ± 0.10 nmol/min/10^9^ cells) than in WT-RS erythrocytes (3.82 ± 0.43 nmol/min/10^9^ cells). GSH-Px activity was reduced in both MT and WT erythrocytes after exercise, but MT-EE activity (2.68 ± 0.03 nmol/min/10^9^ cells) remained significantly higher than WT-EE activity (1.62 ± 0.14 nmol/min/10^9^ cells; Figure 4f). Exhaustive exercise increased glutathione S-transferase (GST) activity in both MT erythrocytes (RS vs. EE, 74.06 ± 3.10 vs. 203.40 ± 3.76 nmol/min/10^8^ cells) and WT erythrocytes (RS vs. EE, 77.36 ± 11.41 vs. 186.34 ± 3.51 nmol/min/10^8^ cells), with GST activity remaining significantly higher in MT-EE compared to WT-EE (Figure 4g). These results indicate that exhaustive exercise reduces erythrocyte antioxidant enzyme activity, and MSTN knockout limits the extent to which antioxidant enzyme activity, especially that of GSH-Px, is affected by exhaustive exercise.

### 3.4. MSTN Knockout Increases Erythrocyte Antioxidant Capacity via GSH

**Figure 5:**
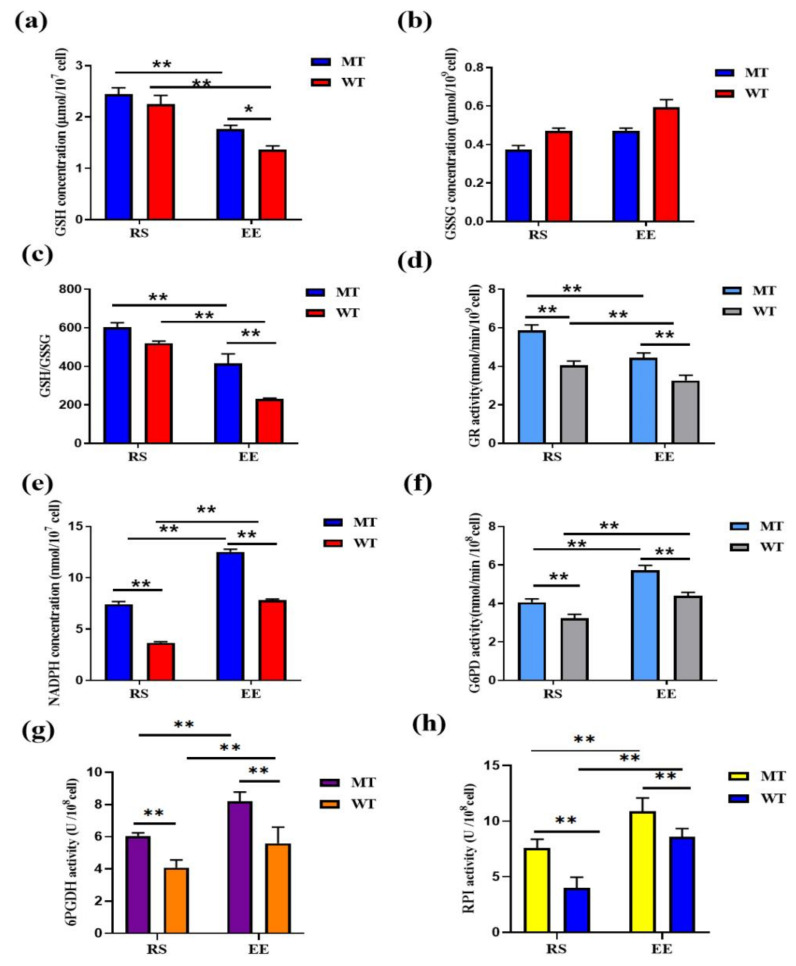
MSTN knockout increases erythrocyte antioxidant capacity via GSH. (**a**) Concentration of GSH; (**b**) Concentration of GSSG; (**c**) Ratio of GSH/GSSG; (**d**) Activity of GR; (**e**) Concentration of NADPH; (**f**) Activity of G6PD; (**g**) Activity of 6PGDH; (**h**) Activity of RPI. MT: MSTN knockout cattle group; WT: Wild type cattle group; RS: Resting state; EE: Exhaustive exercise state; Data presented are means ± SD. One-way ANOVA with post hoc LSD multiple-comparison test. * *p* < 0.05, ** *p* < 0.01.

MT-RS GSH levels (2.44 ± 0.12 μmol/10^7^ cells) were similar to WT-RS levels (2.25 ± 0.16 μmol/10^7^ cells). After exercise, GSH levels decreased rapidly in both MT and WT erythrocytes (RS vs. EE for each cell type), but MT-EE erythrocytes retained more GSH (1.77 ± 0.06 μmol/10^7^ cells) than WT-EE erythrocytes (1.36 ± 0.07 μmol/10^7^ cells; Figure 5a). GSSG concentrations exhibited patterns opposite to GSH levels, and there were no significant differences between groups (MT-RS, 0.37 ± 0.02 μmol/10^9^ cells; WT-RS, 0.47 ± 0.01 μmol/10^9^ cells; MT-EE, 0.47 ± 0.01 μmol/10^9^ cells; and WT-EE, 0.59 ± 0.04 μmol/10^9^ cells; Figure 5b). The GSH/GSSG ratio, an important physiological index of oxidative stress, was higher in MT erythrocytes at both RS (MT vs. WT, 603.36 ± 24.41 vs. 520.04 ± 11.02) and EE (MT vs. WT, 415.70 ± 48.96 vs. 230.86 ± 4.17), with exhaustive exercise affecting the ratio significantly (Figure 5c). GR is the primary enzyme maintaining cellular GSH levels, and MT-RS and MT-EE GR activities (5.87 ± 0.27 and 4.43 ± 0.26 nmol/min/10^8^ cell, respectively) were higher WT-RS and WT-EE GR activities (4.06 ± 0.21 and 3.26 ± 0.28 nmol/min/10^8^ cell, respectively; Figure 5d). Levels of NADPH, which is under the condition that GR needs NADPH to provide reducing power, GSSG is reconverted to GSH to maintain the balance of GSH/GSSG, increased dramatically after exercise in both MT erythrocytes (RS vs. EE, 7.43 ± 0.25 vs. 12.53 ± 0.24 nmol/10^7^ cells) and WT erythrocytes (RS vs. EE, 3.66 ± 0.11 vs. 7.84 ± 0.08 nmol/10^7^ cells). Moreover, MT erythrocytes contained more NADPH than WT erythrocytes at both RS and EE (Figure 5e). NADPH is primarily produced by PPP, and G6PD is the limiting enzyme of PPP. G6PD activity was higher in MT-RS erythrocytes (4.06 ± 0.17 nmol/10^8^ cells) than WT-RS erythrocytes (3.23 ± 0.21 nmol/10^8^ cells), and G6PD remained more active in MT erythrocytes at EE (MT vs. WT, 5.72 ± 0.25 vs. 4.39 ± 0.19 nmol/10^8^ cells; Figure 5f). The rate-limiting enzyme of PPP is 6-phosphogluconate dehydrogenase (6PGDH), and the enzymatic activity of the MT-RS erythrocytes was significantly higher than that of the WT-RS erythrocytes (MT-RS vs. WT-RS, 6.05 ± 0.18 vs. 4.09 ± 0.47 U/10^8^ cells; Figure 5g), while after exhaustive exercise, 6PGDH enzymatic activity in the MT-EE erythrocytes was higher than the WT-EE erythrocytes (MT-EE vs. WT-EE, 8.19 ± 0.57 vs. 5.59 ± 0.99 U/10^8^cells; Figure 5g). Ribulose-5-phosphate isomerase (RPI) is a key enzyme in PPP. The enzyme activity of the MT-RS erythrocytes was higher than that of the WT-RS erythrocytes in resting state (MT-RS vs. WT-RS, 7.59 ± 0.76 vs. 3.91 ± 0.96 U/10^8^ cells; Figure 5h). RPI enzyme activity is up-regulated after exhaustive exercise, and the MT-EE erythrocytes were higher than the WT-EE erythrocytes (MT-EE vs. WT-EE, 10.88 ± 1.19 vs. 8.60 ± 0.72 U/10^8^ cells; Figure 5h). RPI content showed the same trend (Figure S1D). These results suggest that MSTN knockout increases G6PD activity, providing erythrocytes with more NADPH that can contribute to GSH-mediated levels.

### 3.5. MSTN Knockout Affects Erythrocyte Glycolysis

**Figure 6:**
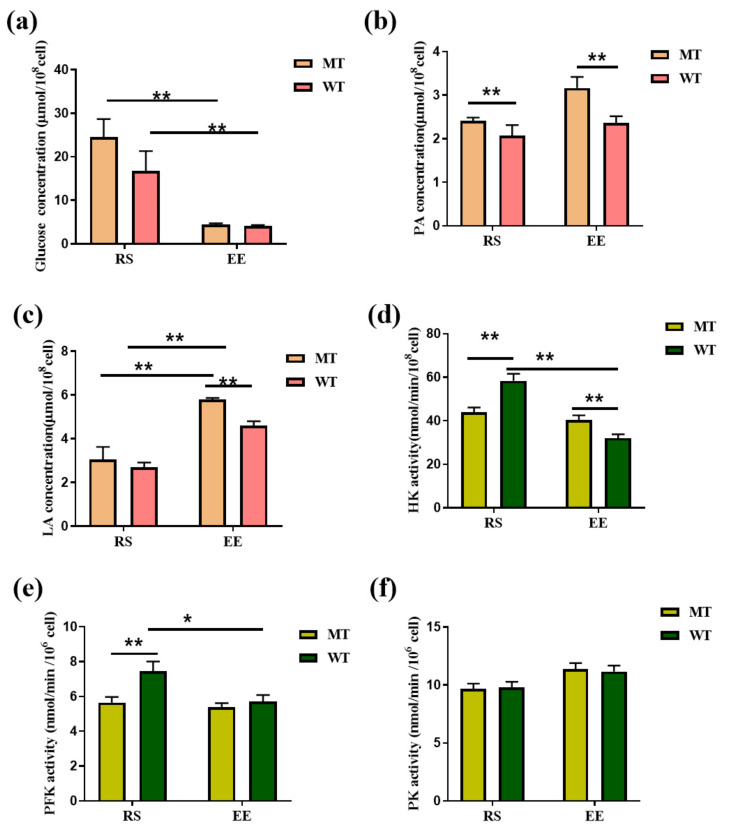
MSTN knockout affected glycolysis of erythrocytes. (**a**) Concentration of glucose; (**b**) Concentration of PA; (**c**) Concentration of LA; (**d**) Activity of HK; (**e**) Activity of PFK; (**f**) Activity of PK. MT: MSTN knockout cattle group; WT: Wild type cattle group; RS: Resting state; EE: Exhaustive exercise state; Data presented are means ± SD. One-way ANOVA with post hoc LSD multiple-comparison test. * *p* < 0.05, ** *p* < 0.01.

Another important route for erythrocyte glucose metabolism is glycolysis. The concentrations of glucose significantly decreased from 24.47 ± 2.43 μmol/10^8^ cell (MT-RS) to 4.47 ± 0.13 μmol/10^8^ cell (MT-EE), and from 16.76 ± 2.62 μmol/10^8^ cell (MT-RS) to 4.07 ± 0.14 μmol/10^8^ cell (WT-EE; Figure 6a). The concentration of pyruvate (PA), a glycolysis end-product, increased in MT erythrocytes after exercise (RS vs. EE, 2.40 ± 0.04 vs. 3.16 ± 0.14 μmol/10^8^ cells), and MT-EE levels were higher than WT-EE levels (2.36 ± 0.08 μmol/10^8^ cells; Figure 6b). LA concentrations were similar in MT-RS erythrocytes (3.06 ± 0.32 μmol/10^8^ cells) and WT-RS erythrocytes (2.69 ± 0.13 μmol/10^8^ cell); LA concentrations increased to 5.78 ± 0.05 μmol/10^8^ cells in MT-EE erythrocytes (RS vs. EE) and 4.61 ± 0.11 μmol/10^8^ cells in WT-EE erythrocytes (RS vs. EE, Figure 6c). The hexokinase (HK) activity was lower in MT-RS erythrocytes (43.95 ± 2.12 nmol/min/10^8^ cells) than WT-RS erythrocytes (40.28 ± 2.19 nmol/min/10^8^ cells). HK activity decreased slightly in MT-EE erythrocytes (40.28 ± 2.19 nmol/min/10^8^ cells; RS vs. EE) and substantially in WT-EE erythrocytes (32.02 ± 1.73 nmol/min/10^8^ cells; RS vs. EE; Figure 6d), and HK activity was significantly higher in MT-EE erythrocytes than in WT-EE erythrocytes. MT phosphofructokinase (PFK) activity did not change significantly between RS (5.64 ± 0.32 nmol/min/10^8^ cells) and EE (5.36 ± 0.24 nmol/min/10^8^ cells; Figure 6e). However, exercise substantially affected WT PFK activity (RS vs. EE, 7.43 ± 0.57 vs. 5.71 ± 0.35 nmol/min/10^8^ cells). Meanwhile, exercise increased PK activity increased in both MT erythrocytes (RS vs. EE, 9.65 ± 0.45 vs. 14.38 ± 0.50 nmol/min/10^8^ cells) and WT erythrocytes (RS vs. EE, 9.80 ± 0.48 vs. 11.17 ± 0.50 nmol/min/10^8^ cells); PK activity was not significantly affected by MTSN (Figure 6f). These results indicate that exhaustive exercise stimulates dramatic changes in erythrocyte glucose metabolism that are influenced by MTSN.

### 3.6. MSTN Knockout Affects Erythrocyte Band 3

**Figure 7:**
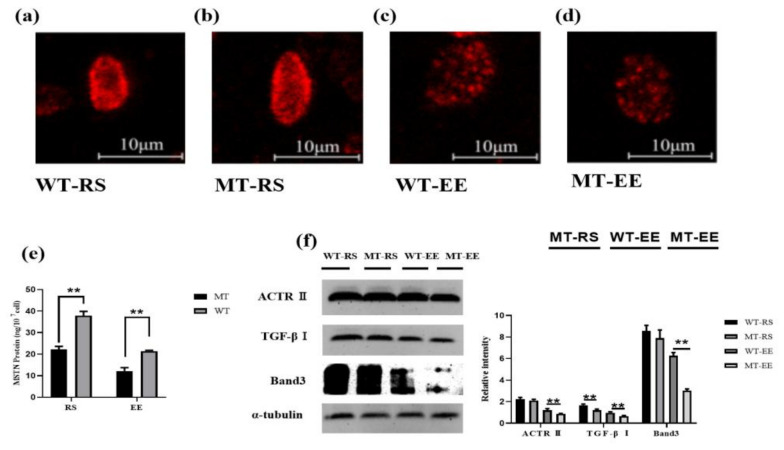
MSTN knockout affected the Band3 protein in the erythrocyte membrane. (**a**) The Band3 protein in the WT-RS group erythrocytes; (**b**) the Band3 protein in the MT-RS group erythrocytes; (**c**) the Band3 protein in the WT-EE group erythrocytes; (**d**) the Band3 protein in the MT-EE group erythrocytes; (**e**) MSTN protein content; (**f**) Western blotting results. MT: MSTN knockout cattle group; WT: Wild type cattle group; RS: Resting state; EE: Exhaustive exercise state; Data presented are means ± SD. One-way ANOVA with post hoc LSD multiple-comparison test. ** *p* < 0:01.

Band 3 is a key protein regulating erythrocyte metabolism. Exhaustive exercise reduced the expression of Band3, there was no difference in Band3 expression between WT-RS and MT-RS, while the expression of Band3 in MT-EE was lower than WT-EE (Figure 7a–d,f). Homozygous (MSTN^−/−^), heterozygotes (MSTN^+/−^, MT) and wild-type (WT) Band 3 were detected in the resting state, and the results showed that homozygous (MSTN^−/−^) Band3 protein expression was lower than that of heterozygotes (MSTN^+/−^, MT) and wild type (WT) (Figure S2A). We used ELISA to detect the expression of MSTN in erythrocytes of MT cattle and WT cattle before and after exhaustive exercise. The results showed that the MSTN protein content of MT-RS cattle was significantly lower than that of the control group (22.102 ± 1.497 vs. 37.9 ± 1.954 ng/10^7^ cell, Figure 7e). And it also showed the same trend after exhaustive exercise (12.159 ± 1.576 vs. 21.42 ± 0.333 ng/10^7^ cell, Figure 7e). Exhaustive exercise can decreased expression of MSTN receptor Act RII and TGF-βRI. Knockout of MSTN did not affect the expression of ActRII, MT-RS and WT-RS showed no difference in ActRII content in resting state, MT-EE and WT-EE showed the same results after exhaustive exercise. In the resting state, the TGF-βRI, MT-RS receptor is significantly higher than WT-RS (Figure 7f). Exhaustive exercise made TGF-βRI expression is decreased, but MT-EE is significantly higher than WT-EE (Figure 7f).

## 4. Discussion

Exhaustive exercise significantly decreased bovine erythrocyte antioxidant capacity and increased oxidative damage, indicating that exhaustive exercise disrupts redox balance in bovine erythrocytes, resulting in a more oxidized state. LA is released from muscles into the blood based on the type, intensity, and duration of exercise, and intensity exercise increases the level of lactic acid in the blood [36]. In this study, lactic acid content increased significantly after exhaustive exercise. Erythrocyte HCT will be increased by exercise [34]. According to the blood routine results of this study, we found that red blood cell HCT showed an upward trend after exhaustive exercise. Exhaustive exercise decreased membrane levels of FA, especially SFA and MUFA, resulting in decreased membrane fluidity. However, C20:4n-6 levels, which correlate with erythrocyte oxalate levels [37], C20:4n-6 gives rise to prostaglandins and leukotrienes that are proinflammatory in a context-dependent fashion [38]. Surprisingly, SOD, POD, and GSH-Px activities decreased after exercise, as did GSH levels. However, exhaustive exercise did not significantly decrease protein sulfhydryl levels, despite that GSH functions to keep protein thiols in a reduced state, thereby preserving erythrocyte function. Our results suggest that, in an attempt to quickly restore GSH levels after exhaustive exercise, erythrocyte glycolysis is inhibited and the PPP is activated, thereby increasing NADPH synthesis and accelerating GSH transformation.

Under normal conditions, erythrocytes maintain redox balance, and MSTN knockout does not affect erythrocyte oxidative damage. However, exhaustive exercise disrupts erythrocyte redox balance, resulting in oxidative damage; it is under these conditions that MSTN knockout has evident effects. Compared with WT erythrocytes, MT erythrocytes harbor less ROS (H_2_O_2_ and O_2_^−^), oxidative damage, MDA, PC, and nonprotein sulfhydryls. Additionally, the levels of C20:4n-6, a proinflammatory factor, was significantly increased in MT erythrocytes after exercise, although the mechanism underlying this increase remains unclear. According to the measured activities of SOD, CAT, and POD, MSTN has little effect on erythrocyte enzymatic antioxidants. Instead, MSTN knockout enhances ROS scavenging via the nonenzymatic antioxidant GSH. There are two sources of GSH in erythrocytes: GSH synthesis from amino acids, and the conversion of GSSG to GSH by GR. NADPH, which is produced by G6PD, is a crucial CR cofactor [39]. The high GSH concentration observed in MT erythrocytes is explained by the high GR and G6PD activities in these cells, as increased G6PD activity within the PPP provides erythrocytes with additional NADPH. Overall, MSTN primarily affects erythrocyte antioxidant capacity via the PPP and, consequently, via GSH levels.

In mature erythrocytes, energy production depends on glycolysis, which uses glucose as a substrate. Following glucose uptake from the plasma, HK converts glucose to G6P, of which ~5–10% enters the PPP; the remaining 90% is metabolized via glycolysis to PA and LA. HK, PFK, and PK are the key glycolytic enzymes, and decreases in their activities result in diminished glycolysis rates in erythrocytes. Band 3 is the most abundant protein in the erythrocyte membrane and, in conjunction with Band 4.1, Band 4.2, ankyrin, and spectrin, maintains the structural stability of the membrane [40]. Band 3 also regulates blood HCO^3−^/CO^2−^ metabolism [41] and erythrocyte glucose metabolism [42]. In the study, Bnad3 expression decreased after exhaustive exercise, which was associated with decreased Band3 levels and increased aggregation, consistent with previously observed changes in Band3 in human erythrocytes [43]. The content of end-products of glycolysis, pyruvate and lactate, increased after MSTN knockout exercise. Knockout of MSTN reduced the activity of the glycolytic rate-limiting enzyme PFK. Band 3 is a transmembrane protein of erythrocytes, the cytoplasmic portion of which regulates the pentose phosphate pathway (PPP) by competitively binding to the central lumen of the deoxygenated hemoglobin β chain and some glycolytic enzymes [44]. Glycolytic enzymes bind to Band 3 proteins with greater advantage. When the glucose molecule enters the erythrocyte, it turns to the PPP because glycolytic enzymes have been involved with the Band 3 protein, which reduces cytosolic glycolysis within the erythrocyte [44,45].

## 5. Conclusions

Erythrocytes are of interest regarding exercise-induced oxidative damage due to their important roles in free radical scavenging and redox balance maintenance in the body. Here, we showed that MSTN knockout alleviates exercise-induced oxidative stress by increasing the efficiency of the PPP, thereby increasing erythrocyte GSH content and antioxidant potential.

## Figures and Tables

**Table 1:** Effect of exhaustive exercise on hematological parameter of RBCs in different group.

	MT-RS	WT-RS	MT-EE	WT-EE
RBC (10^12/L)	7.72 ± 0.77	7.82 ± 0.43	7.87 ± 0.75	7.48 ± 0.46
RDW (fL)	10 ± 1.0	10.0 ± 1.04	9.67 ± 1.03	9.58 ± 1.08
HCT (%)	3403 ± 299	3388 ± 140	3584 ± 236	3506 ± 249
MCV (fL)	43.19 ± 2.2	45.7 ± 3.1	43.3 ± 2.2	45.2 ± 3.0
MCH (Pg)	14.71 ± 0.79	14.51 ± 0.93	14.78 ± 0.85	14.42 ± 1.04
MCHC (g/L)	31.78 ± 0.67	32.07 ± 0.27	31.5 ± 0.55	31.93 ± 0.47
HGB (g/dl)	111.2 ± 8.63	110.88 ± 6.79	109.46 ± 11.46	107.72 ± 10.59

RBC: red blood cell count; RDW: red cell distribution width; HCT: hematocrit; MCV: mean corpuscular volume; MCH: mean corpuscular hemoglobin; MCHC: mean corpuscular hemoglobin concentration; HGB: hemoglobin.

## Data Availability

The data generated and analyzed during this study are available upon reasonable request from the corresponding author.

## Supplementary Materials

The following supporting information can be downloaded at: https://www.mdpi.com/article/10.3390/ani12070927/s1, Table S1: Body weight of cattle at different age (kg); Table S2: Body length of cattle at different age (cm); Table S3: Wither length of cattle at different age (cm). Figure S1: MSTN knockout affects erythrocyte FA changes after exhaustive exercise. (**A**) Total fatty acid concentration of erythrocytes; (**B**) SFA and UFA concentration; (**C**) MUFA 2 and PUFA concentration; (**D**) Concentration of RPI; (**E**) Content of fatty 3acids in n-3 and n-6 PUFA. MT: MSTN knockout cattle group; WT: Wild type cattle group; RS: Resting state; EE: Exhaustive exercise state; Data presented are means ± SD. One-way ANOVA with post hoc LSD multiple-comparison test. ∗ *p* < 0.05, ∗∗ *p* < 0.01.; Figure S2: Full original blots used for Figure 7f, each blot membrane was cut based on the standard band positions and then incubated with the appropriate antibodies. The bands in the article are marked in a red frame, and the bands not included in the article are marked in green frame. (**A**) Band3 protein expression level; homozygous. (MSTN^-/-^), heterozygous (MSTN^+/-^), wild type; (**B**) Full original blots used for Figure 7f. Each blot membrane was cut based on the standard band positions and then incubated with the appropriate antibodies. The bands in the article are marked in red frame, and the bands not include in the article are marked in green frame.
