# Supplementary material for: Myostatin Knockout Limits Exercise-Induced Reduction in Bovine Erythrocyte Oxidative Stress by Enhancing the Efficiency of the Pentose Phosphate Pathway
Source: Animals (Basel). 2022 Apr 4;12(7):927. doi: 10.3390/ani12070927 (PMC8996956; doi:10.3390/ani12070927)
Supplement: Supplementary file 1 [file animals-12-00927-s001.zip › animals-1625221-supplementary.pdf]

animals

Supplementary Materials

Myostatin Knockout Limits Exercise-Induced Reduction in Bovine Erythrocyte Oxidative Stress by Enhancing the Efficiency of the Pentose Phosphate Pathway

Lin Zhu, Chunling Bai, Xueqiao Wang, Zhuying Wei, Mingjuan Gu, Xinyu Zhou, Guanghua Su, Xuefei Liu, Lei Yang * and Guangpeng Li *

State Key Laboratory of Reproductive Regulation and Breeding of Grassland Livestock, College of Life Science, Inner Mongolia University, Hohhot 010021, China; zhulinynacxhs@163.com (L.Z.); chunling1980_0@163.com (C.B.); wangxueqiao05@126.com (X.W.); weizhuying2008@126.com (Z.W.); gmj0119@yeah.net (M.G.); zhouxinyufjb@163.com (X.Z.); suguanghua0707@163.com (G.S.); liuxuefei1006@126.com (X.L.)

* Correspondence: mrknowall@126.com (L.Y.); gpengli@imu.edu.cn (G.L.)

Table S1. Body weight of cattle at different age (kg).

	MT	WT
3 months old	98.34 ± 13.36	96.96 ± 7.45
6 months old	180.83 ± 20.64	152.90 ± 11.66
9 months old	220.34 ± 19.89	183.55 ± 18.66
12 months old	269.40 ± 16.22	249.08 ± 17.16
15 months old	327.15 ± 43.13	293.27 ± 29.76

MT:MSTN gene- editing cattle; WT: wild type cattle.

Table S2. Body length of cattle at different age (cm).

	MT	WT
3 months old	94.20 + 7.35	91.10 ± 2.61
6 months old	104.75 ± 2.88	100.13 ± 5.05
9 months old	113.39 ± 3.96	112.57 ± 1.27
12 months old	126.50 ± 3.50	121.71 ± 2.64
15 months old	133.73 ± 5.20	128.03 ± 4.40

MT:MSTN gene- editing cattle; WT: wild type cattle.

Table S3. Wither length of cattle at different age (cm).

	MT	WT
3 months old	89.85 ± 3.92	89.85 ± 3.92
6 months old	103.88 ± 2.16	103.88 ± 2.16
9 months old	112.52 ± 2.19	112.52 ± 2.19
12 months old	115.32 ± 1.89	115.32 ± 1.89
15 months old	120.46 ± 1.83	120.46 ± 1.83

MT:MSTN gene- editing cattle; WT: wild type cattle.

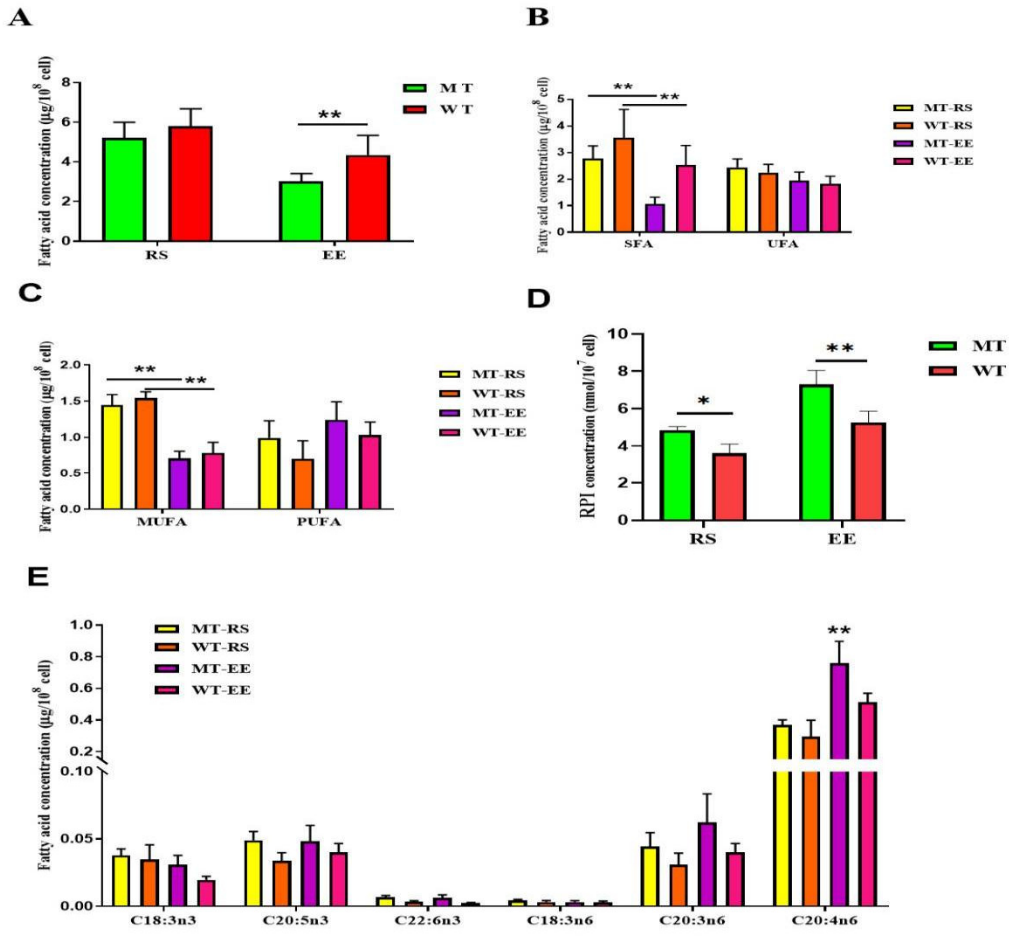

Figure S1. MSTN knockout affects erythrocyte FA changes after exhaustive exercise. (**A**) Total fatty acid concentration of erythrocytes; (**B**) SFA and UFA concentration; (**C**) MUFA 2 and PUFA concentration; (**D**) Concentration of RPI; (**E**) Content of fatty 3acids in n-3 and n-6 PUFA. MT: MSTN knockout cattle group; WT: Wild type cattle group; RS: Resting state; EE: Exhaustive exercise state; Data presented are means ± SD. One-way ANOVA with post hoc LSD multiple-comparison test. $* \, p < 0.05$, $** \, p < 0.01$.

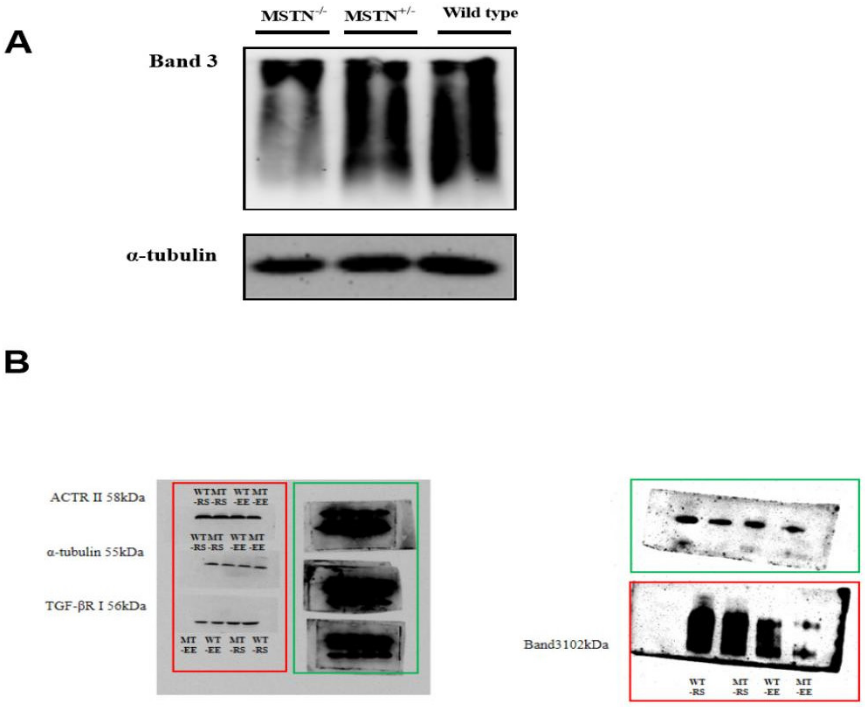

Figure S2. Full original blots used for Figure 7f, each blot membrane was cut based on the standard band positions and then incubated with the appropriate antibodies. The bands in the article are marked in a red frame, and the bands not included in the article are marked in green frame. (**A**) Band3 protein expression level; homozygous. ($MSTN^{-/-}$), heterozygous ($MSTN^{+/-}$), wild type; (**B**) Full original blots used for Figure 7f. Each blot membrane was cut based on the standard band positions and then incubated with the appropriate antibodies. The bands in the article are marked in red frame, and the bands not include in the article are marked in green frame.
